# Human dental stem cells suppress PMN activity after infection with the periodontopathogens *Prevotella intermedia* and *Tannerella forsythia*

**DOI:** 10.1038/srep39096

**Published:** 2016-12-15

**Authors:** Cathleen Hieke, Katja Kriebel, Robby Engelmann, Brigitte Müller-Hilke, Hermann Lang, Bernd Kreikemeyer

**Affiliations:** 1 Department of Operative Dentistry and Periodontology, University Medical Center Rostock, Rostock, Germany; 2 Institute of Immunology, University Medical Center Rostock, Rostock, Germany; 3 Institute of Medical Microbiology, Virology and Hygiene, University Medical Center Rostock, Rostock, Germany

## Abstract

Periodontitis is characterized by inflammation associated with the colonization of different oral pathogens. We here aimed to investigate how bacteria and host cells shape their environment in order to limit inflammation and tissue damage in the presence of the pathogen. Human dental follicle stem cells (hDFSCs) were co-cultured with gram-negative *P. intermedia* and *T. forsythia* and were quantified for adherence and internalization as well as migration and interleukin secretion. To delineate hDFSC-specific effects, gingival epithelial cells (Ca9-22) were used as controls. Direct effects of hDFSCs on neutrophils (PMN) after interaction with bacteria were analyzed via chemotactic attraction, phagocytic activity and NET formation. We show that *P. intermedia* and *T. forsythia* adhere to and internalize into hDFSCs. This infection decreased the migratory capacity of the hDFSCs by 50%, did not disturb hDFSC differentiation potential and provoked an increase in IL-6 and IL-8 secretion while leaving IL-10 levels unaltered. These environmental modulations correlated with reduced PMN chemotaxis, phagocytic activity and NET formation. Our results suggest that *P. intermedia* and *T. forsythia* infected hDFSCs maintain their stem cell functionality, reduce PMN-induced tissue and bone degradation via suppression of PMN-activity, and at the same time allow for the survival of the oral pathogens.

The oral microbiome contains hundreds of different species forming complex multi-species biofilms^1^,^2^. The existence of some species and composition of such biofilms are potential indicators to distinguish between health and disease^2^,^3^,^4^,^5^. Especially a shift of species to gram-negative anaerobic bacteria, e.g. *Aggregatibacter actinomycetemcommitans, Fusobacterium nucleatum, Porphyromonas gingivalis, Prevotella intermedia* and *Tannerella forsythia*, is associated with progressive periodontitis^6^,^7^,^8^,^9^. Periodontitis is an inflammatory disease of the periodontium induced by bacteria and is characterized by progressive irreversible loss of supportive tissue and finally tooth loss. The oxygen level is decreased in periodontal pockets allowing those anaerobic species to colonize^10^,^11^ and disrupt host cell homeostasis^12^. With increasing access to deeper tissues, periodontal pathogenic bacteria get in contact with various local cell types beside epithelial cells, including different progenitor cells. *In vitro* studies documented increasing migration of mesenchymal stem cells under hypoxic conditions^13^. Furthermore, bacterial LPS ambivalently affects human dental pulp stem cell migration, enhancing at 1μg/ml, inhibiting at 10μg/ml^14^. *P. gingivalis* LPS inhibits periodontal ligament stem cell osteoblastic differentiation^15^. Nonetheless, bacterial infection with viable microorganisms presumably has diverse effects on host cells.

As part of tissue repair mechanisms, investigation on stem cell-bacteria interaction is important for better understanding of periodontal disease progression. The direct interaction between bacteria and host cells stimulates immune response in terms of secretion of a wide range of cytokines^16^,^17^,^18^,^19^. A major recruiting factor for phagocytic polymorphonuclear leukocytes (PMNs) is interleukin-8 which provokes PMN accumulation^20^,^21^, further tissue damage and progression of periodontitis^22^. Human mesenchymal stem cells and gingival epithelial cells were shown to secrete IL-8 after infection with *F. nucleatum* and *P. gingivalis in vitro*^23^. Additionally, *Prevotella intermedia* LPS provokes IL-8 secretion in human dental pulp fibroblasts^24^ and interacts with KB cells^25^, a HeLa derived cell line. Interaction of *T. forsythia* with Ca9-22 or KB cells was also previously investigated^26^.

Taken together it can be hypothesized that host cells, including progenitor stem cells, pathogenic bacteria and primary defense cells like PMNs, engage in interactions in the periodontal pocket. The nature of this interplay could determine periodontitis disease outcome. Therefore, the aim of this study was to analyze how oral pathogenic bacteria influence oral stem cells and oral epithelial cells *in vitro* with a focus on effects they might execute towards PMNs. Our experimental setup was designed to achieve a deeper insight in immunological mechanisms in this complex infection model. In this study, the interaction between periodontal pathogens and human dental follicle stem cells (hDFSCs) was investigated. To understand the specific role of these stem cells, results were compared to the gingival epithelial cell line (Ca9-22). As stem cells have immunomodulatory and tissue regenerating function^27^, we aimed to demonstrate the effects hDFSCs primed by initial bacterial pathogen contact have on PMNs, which represent a major factor in periodontal inflammation and tissue destruction.

*P. intermedia* and *T. forsythia* were chosen for infection as only little is known about those species compared to the well-studied species *P. gingivalis, A. actinomycetemcommitans* and *F. nucleatum*. Both species are tightly associated with periodontal disease^28^,^29^, although underrepresented in the current literature.

## Results

### Anaerobic cultivation of hDFSCs

Survival of hDFSCs under anaerobic conditions was observed over 72 h via trypan blue staining, metabolic activity using MTS and expression of stem cell marker.

**Figure 1:**
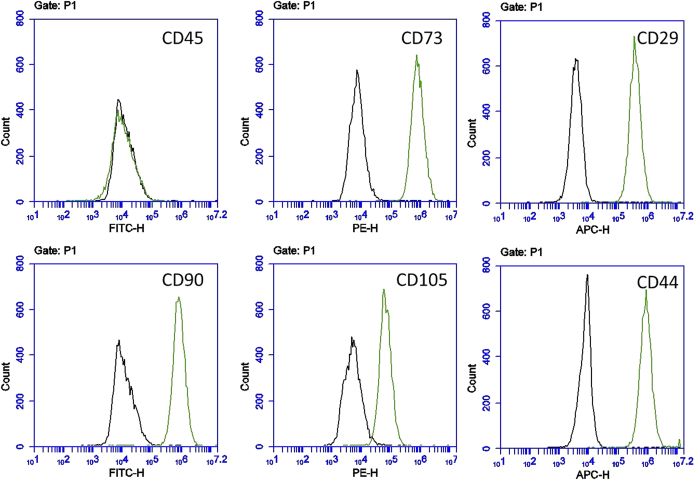
Surface marker expression of hDFSCs. Representative FACS analysis of the stem cell surface marker on hDFSCs after incubation under aerobic and anaerobic conditions. The black line refers to the isotype control, green describes the distinct antibody binding on the stem cell surface marker.

Under anoxic conditions cell count is reduced to 75% after 24 h, 60% after 48 h and 30% after 72 h compared to aerobic cultivated cells. Thereby, the metabolic activity is reduced. After standardization to the diminished cell count metabolic activity is comparable to aerobic cultivated hDFSCs (see Supplementary Fig. S1). Expression of CD73, CD29, CD90, CD105 and CD44 (CD45-negative) was stable over 72 h of anaerobic incubation (Fig. 1, see Supplementary Tab. S1 for median fluorescence intensity data).

### Oral bacteria invade hDFSCs

Adherence and internalization are major steps in host invasion and pathogenesis. The direct interaction between undifferentiated hDFSCs and oral species was examined in terms of adherence and internalization after 2 h of anaerobic co-culture. The differentiated gingival epithelial cell line Ca9-22 was used as control (MOI = 100).

**Figure 2:**
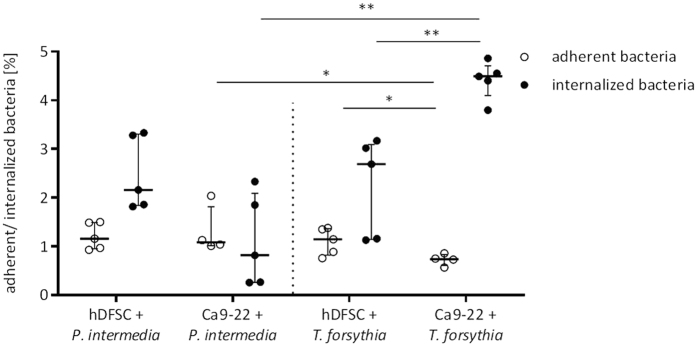
Direct interaction of *P. intermedia* and T. *forsythia* with human cells. HDFSCs and Ca9-22 were infected with *P. intermedia* or *T. forsythia* under anaerobic conditions for 2 h. Adherent and internalized bacteria were quantified. Adherent bacteria were related to reference bacteria present in the inoculum, and internalized bacteria were related to adherent bacteria. Results are displayed as median ± interquartile range, *p < 0.05, **p < 0.01 (Mann-Whitney U test), n ≥ 4.

As shown in Fig. 2, *P. intermedia* adheres equally well to hDFSCs and Ca9-22 cells (about 1% of the inoculum). However, the percentage of *P. intermedia* internalized into hDFSCs is higher compared to values of the *P. intermedia*-Ca9-22 interaction. *T. forsythia* has a distinct phenotype in this assay. Adherence to hDFSCs is more pronounced compared to Ca9-22 cells, whereas internalization into Ca9-22 was significantly higher for *T. forsythia*. Together, these data show that both species are able to adhere and internalize into hDFSCs as well as Ca9-22 with distinct phenotypes (Fig. 2).

### Bacterial infection had neither impact on stem cell marker surface expression nor the differentiation potential

Among others, characteristics of stem cells are the expression of typical stem cell surface markers and the potential to differentiate into various cell lineages depending on stimulus. Thus, it was mandatory to investigate alterations in surface marker display and differentiation under the infection situation described in the previous result section.

**Figure 3:**
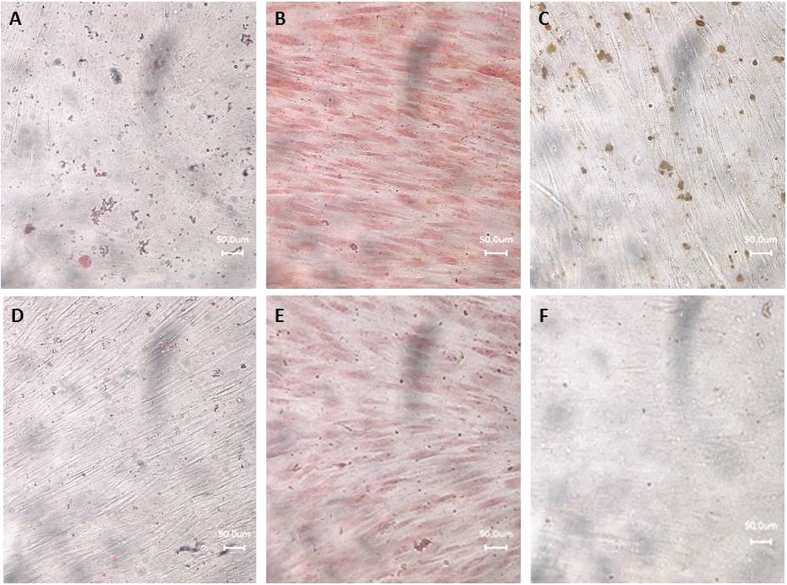
Differentiation potential of infected hDFSCs. Representative microscopic photograph of infected hDFSCs after 21 days of differentiation stimulation. Adipogenic (**A**), chondrogenic (**B**) and osteogenic (**C**) differentiation stain. (**D**) to (**F**) are infected control cells with cell culture medium.

In summary, after infection of hDFSCs with *P. intermedia* or *T. forsythia* (MOI = 100) no alternation in stem cell surface marker expression (positive for CD73, CD29, CD90, CD105 and CD44; negative for CD45) under anaerobic atmosphere and differentiation potential to adipogenic, chondrogenic and osteogenic fate was detected compared to uninfected cells (Fig. 3, see Supplementary Tab. S2 for median fluorescence intensity data of infected hDFSCs).

### Bacterial infection and anaerobic condition reduce hDFSC migration

The migration ability of adherent cells is important in the context of tissue repair and cell repopulation during wound healing^30^,^31^. It was shown, that LPS and hypoxia alter migration of stem cells *in vitro*^13^,^14^,^32^. Consequently, migration activity of hDFSCs was quantified under aerobic and anaerobic conditions with or without bacterial infection (MOI = 100). This phenotype was monitored every 4 h post-infection up to 24 h post infection of co-culture in 24 well plates.

**Figure 4:**
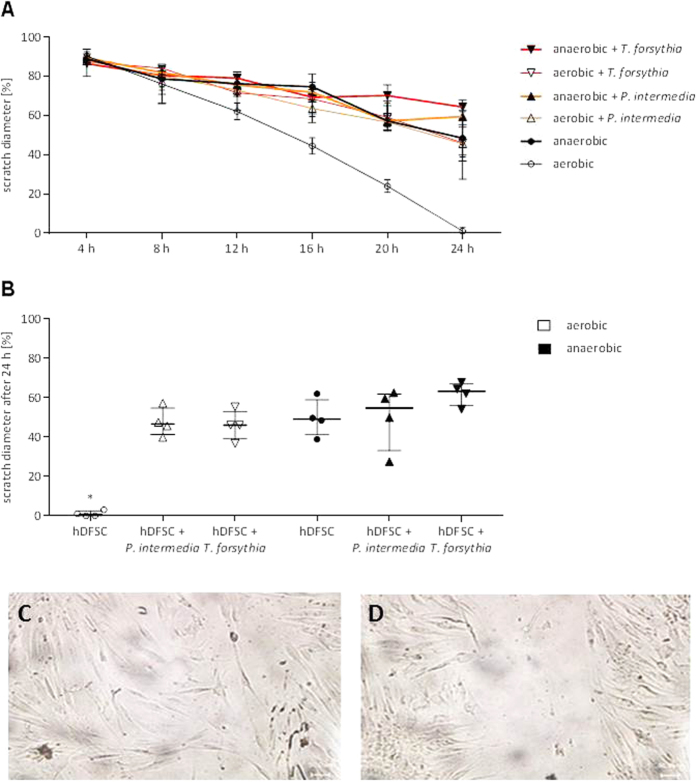
Migration of hDFSCs after 24 h. HDFSCs were incubated under aerobic or anaerobic conditions and in parallel infected with *P. intermedia* or *T. forsythia*. Uninfected hDFSCs were used as control. Migration was analyzed in a scratch assay, while the scratch diameter was measured each 4 h up to 24 h. The initial scratch diameter was defined as 100%. The migration dynamic of infected and uninfected hDFSCs is observed over 24 h, results are displayed as median ± standard deviation (**A**) and final results are presented after 24 h of incubation. Results are displayed as median ± interquartile range, *p < 0.05 (Mann-Whitney U test), n = 4 (**B**). Representative microscopic photograph of the migrating HDFSCs after 24 h of aerobic (**C**) and anaerobic (**D**) incubation.

Under aerobic conditions hDFSCs efficiently migrate to completely heal the scratch. An anaerobic atmosphere reduces this capacity by 50%. Under aerobic conditions infection of hDFSCs with both *P. intermedia* and *T. forsythia* leads to similarly reduced migration capacities. Infection with *T. forsythia* under oxygen limitation further impaired cellular migration capacity, however, not reaching significance levels (Fig. 4). Of note, an expected additive effect of infection and anaerobic stress concerning migration of hDFSCs was not observed. The migration of hDFSCs is reduced after anoxic stress and infection. *P. intermedia* and *T. forsythia* infection of hDFSCs suppress migration and thus scratch healing. This effect is more significant compared to anaerobic conditions.

### Bacterial infection induces interleukin secretion by hDFSCs

As response to bacterial infection human cells are able to secrete a wide range of cytokines with pro- and/or anti-inflammatory effects to influence their environment. Thus, after infection of hDFSCs or Ca9-22 with *P. intermedia* or *T. forsythia* accumulation of interleukins IL-6, IL-8 and IL-10 in the supernatant was quantified after 2 h, 4 h and 24 h of anaerobic co-culture (MOI = 100).

**Figure 5:**
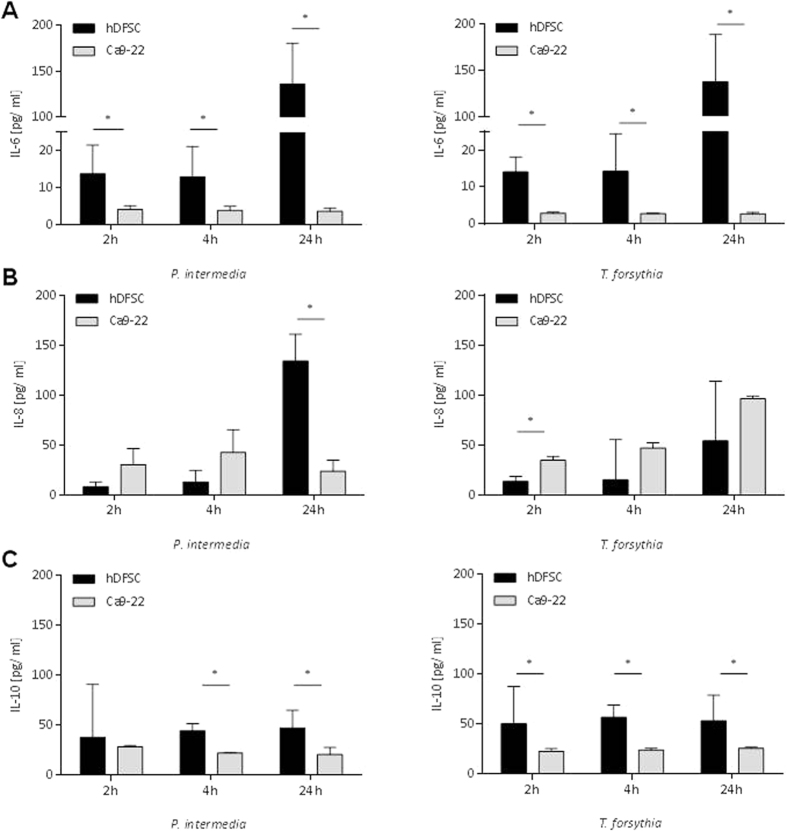
Secretion of IL-6, IL-8 and IL-10 after infection of human cells. HDFSCs and Ca9-22 were infected with *P. intermedia* or *T. forsythia* under anaerobic conditions. IL-6 (**A**), IL-8 (**B**) and IL-10 (**C**) levels were quantified via ELISA after 2 h, 4 h and 24 h from the supernatant of infected cells with *P. intermedia* (left) and *T. forsythia* (right). Values from uninfected hDFSC controls matching the experimental time points were subtracted beforehand. Results are displayed as median ± interquartile range, *p < 0.05 (Mann-Whitney U test), n = 4.

Infection of hDFSCs with *P. intermedia* or *T. forsythia* results in increasing IL-6 concentration in the supernatant after 24 h. In contrast, the gingival epithelial cell line Ca9-22 does not respond to *P. intermedia* or *T. forsythia* infection with increased IL-6 secretion emphasizing the unexpected and obviously specific phenotype of the infection-responsive undifferentiated hDFSCs. (Fig. 5A).

A significant accumulation of IL-8 by infected hDFSCs after *P. intermedia* and *T. forsythia* infection was exclusively detected after 24 h. *P. intermedia* infected hDFSCs show a highly significant increase compared to infected Ca9-22 cells, which are rather unresponsive in terms of IL-8 secretion. The Ca9-22 cell response to *T. forsythia* infection is comparable to hDFSCs with only a marginally significant difference 2 h post-infection (Fig. 5B).

No time-dependent accumulation of anti-inflammatory IL-10 was detected for either cell type infected with *P. intermedia* or *T. forsythia*. Of note, the general levels of accumulated IL-10 in infected hDFSCs are higher, reaching significance for almost all measurement time points. In general, hDFSCs are more responsive towards infection with *P. intermedia* or *T. forsythia* (Fig. 5C).

Together, *P. intermedia* and *T. forsythia* are capable of provoking hDFSCs to secrete inflammatory cytokines like IL-6 and IL-8 in similar or higher concentrations compared to gingival epithelial cells. However, this pro-inflammatory response appears to be counterbalanced by constant IL-10 level.

### Infected hDFSCs can reduce chemotaxis of PMN

Cytokines, e.g. interleukins, are secreted by cells to influence their direct environment. PMNs are recruited to infection sites following a chemical gradient of especially IL-8, which can be produced by hDFSCs, as shown in the previous section. The attraction potential of *P. intermedia* and *T. forsythia* after infection of hDFSCs was addressed in a transwell migration assay. To this end, migration of PMN towards sterile filtered supernatants of hDFSCs and/or bacteria was quantified after 2 h of aerobic incubation in cell culture medium.

**Figure 6:**
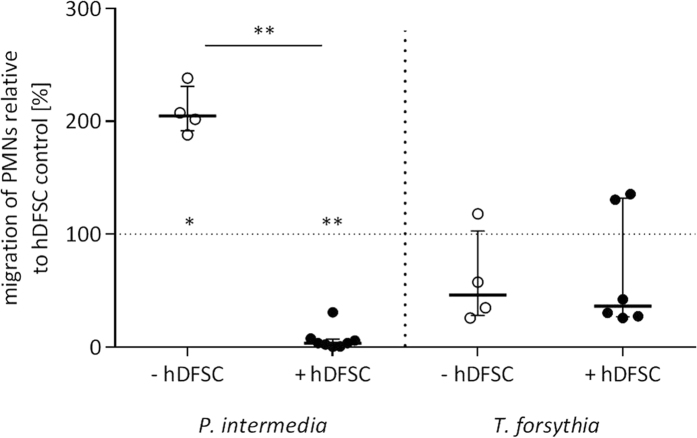
Chemotactic behavior of PMNs towards infected hDFSCs. Migration of PMNs was assessed via transwell assay. After 24 h sterile filtered supernatants of *P. intermedia* and *T. forsythia* in cell culture medium with/without hDFSCs were used as chemo attractants in the lower wells. PMNs in fresh cell culture medium without serum were added to the insert. After 2 h of aerobic incubation the count of PMNs migrated into the lower compartment was determined. Migration of PMNs in the presence of non-infected hDFSCs was set to 100% (dashed line). Results are displayed with median ± interquartile range. *p < 0.05, **p < 0.01 (Mann-Whitney U test), n ≥ 4.

To evaluate the different culture influences, PMN migration towards untreated hDFSCs is determined and set to 100%. The bacterial culture supernatant of *P. intermedia* leads to high level attraction and migration of PMNs (209.1%). Presence of hDFSCs in the initial interaction with *P. intermedia* leads to an almost abolished PMN chemotaxis (6.8%), which implies a suppressive effect of hDFSCs in co-culture with the microorganism. *T. forsythia* supernatants alone and after hDFSC infection lead to 50% reduction of PMN movement towards the infection stimulus. Thus, hDFSCs are able to influence the chemotactic attraction potential of bacteria towards PMNs in a species specific manner (Fig. 6).

### Infected hDFSCs reduce phagocytic activity of PMNs

As hDFSCs secrete several cytokines and influence PMN chemotaxis, the influence on phagocytosis, an important component of the innate immune response of PMNs, was next characterized.

The phagocytic activity of PMNs was determined by quantification of bacteria in the supernatant after 2 h of incubation in presence and absence of hDFSCs. To prevent bacteria from internalization into hDFSCs, the cytoskeleton inhibitor of actin polymerization Latrunculin was added to hDFSCs. The inhibitor was removed prior to bacterial infection in order to allow undisturbed PMN activity at later stages of the experiment. This treatment completely abolished bacterial internalization (data not shown). After 2 h of anaerobic incubation with PMNs bacterial counts were assessed.

**Figure 7:**
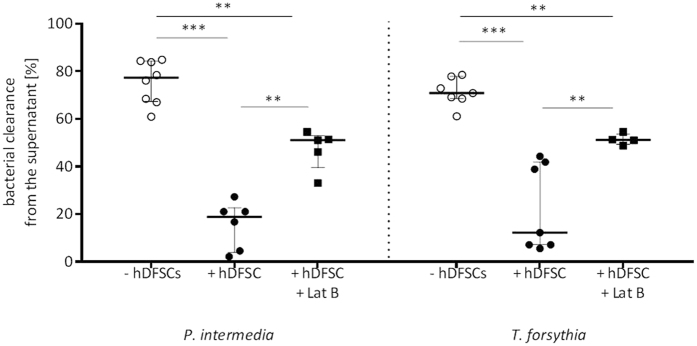
Phagocytic activity of PMNs against oral bacteria. Phagocytic activity is shown as bacterial clearance from the supernatant of *P. intermedia* or *T. forsythia* with hDFSCs relative to the control of bacteria only. Results are displayed with median ± interquartile range. **p < 0.01, ***p < 0.001 (Mann-Whitney U test), n ≥ 4.

First, the number of bacteria used for infection was considered the maximum number that could be cleared and was therefore considered a 100% clearance. Accordingly, within two hours, PMNs eliminated 75.5% of inoculated *P. intermedia* and 71.3% of *T. forsythia*. Presence of hDFSCs led to a significantly reduced bacterial clearance efficiency of the PMNs (15.5% clearance of *P. intermedia*, 22.5% clearance of *T. forsythia*). Inhibition of bacterial internalization into hDFSCs results in moderate but still significant reduction of clearance (47.2% clearance of *P. intermedia*, 51.4% clearance of *T. forsythia*). Thereby, the protective effect of hDFSCs against PMN induced bacterial clearance has two distinguishable levels. First, a direct influence of hDFSCs on PMN activity, and second, internalization of bacteria into hDFSCs further reduce the number of available bacterial for PMN mediated clearance (Fig. 7).

### Infected hDFSCs reduce NET formation of PMN

Another important feature of PMN defense is the formation of neutrophil extracellular traps in response to harmful signals. PMNs were stimulated with supernatants of infected hDFSCs, bacteria or cells for 180 min, and NET formation was quantified with respect to extracellular DNA content. As positive control PMNs were stimulated with glucose oxidase.

**Figure 8:**
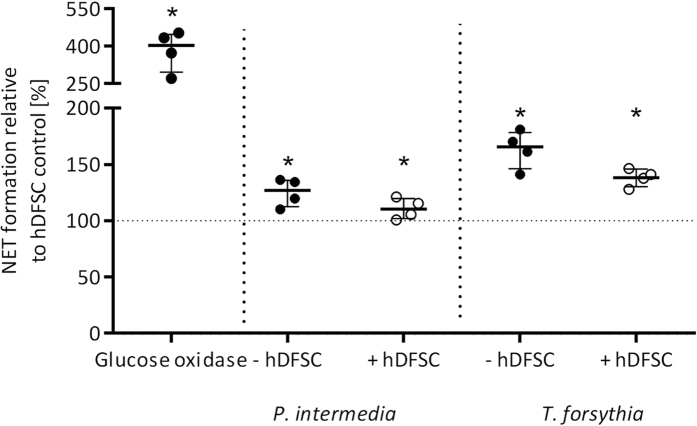
NET formation of PMNs against oral bacteria. NETs were quantified via quantification of extracellular DNA. HDFSCs and *P. intermedia* or *T. forsythia* were used as stimuli for PMNs. After 180 min, extracellular DNA was quantified and uninfected hDFSCs were set to 100%. Results are displayed with median ± interquartile range. *p < 0.05 (Mann-Whitney U test) significance to hDFSC control, n = 4.

Extracellular DNA content of PMNs challenged with hDFSCs in cell culture medium is set to 100%. Bacterial presence increases DNA release further. Nevertheless, the stimulus is higher using bacteria without stem cells (143.0% for *P. intermedia*, 186.1% for *T. forsythia*). Under glucose oxidase stimulation of PMN maximum NET formation was observed in the supernatant. In consequence, hDFSCs apparently reduce NET formation of PMNs after bacterial infection (Fig. 8).

## Discussion

The major focus of this study was to investigate the trilateral interaction of periodontopathogenic bacteria, dental stem cells and PMN activity. This constellation occurs during active periodontitis in patients, and thus we aimed to elucidate if *in vitro* results could at least partially explain clinical phenotypes in periodontitis. This inflammatory disease is characterized by bacterial biofilms in periodontal pockets, a sustained inflammatory reaction as innate immune response to bacterial presence, hyper-immune activation, osteoclast activation, tissue and bone resorption and finally tooth loss.

In this study, we demonstrated different effects of infection of human dental follicle stem cells with either *P. intermedia* or *T. forsythia*. With respect to the anaerobic bacterial species, *P. intermedia* and *T. forsythia,* and low partial pressure of oxygen in periodontal pockets^10^,^11^,^30^,^31^, an anaerobic *in vitro* model described by Kriebel *et al*.^23^ was applied. The consequences of anoxic stress towards the dental stem cells (hDFSCs) were analyzed. As previously shown by Kanafi and colleagues migration and proliferation of human dental pulp stem cells (SHEDs and DPSCs) were increased under hypoxic conditions of 2.3% O_2_ und 5% CO_2_ compared to normoxic conditions^33^. Even 3% of oxygen stimulated migration of mesenchymal bone marrow stem cells^13^,^34^,^35^. In the experimental system studied here we show that anoxia lead to significantly lower migration, and no proliferation was observed. The infection of hDFSCs also reduced migration, but had no additive effect on migration. Interestingly, LPS from *P. gingivalis* was described as a positive stimulus for dental stem cell migration, although no increase in interleukin levels was shown^32^. Nonetheless, stimulation of cells with bacterial components may differ from an infection with vital bacteria, a note that favors our experimental setup. In co-culture of primary human keratinocytes with *P. gingivalis* or *F. nucleatum* a species specific delay in scratch closure was demonstrated in aerobic condition^36^. Thus, migration activity is influenced by various factors, among them cell type, microbial stimulation modus and culture conditions.

Adherence and internalization are key steps in host infection. For this purpose, various taxa specific adhesion molecules are expressed by oral species^37^. It was already shown that different oral pathogens, e.g. *P. gingivalis, F. nucleatum* and *A. actinomycetemcommitans*, are able to enter different host cell types^18^,^38^,^39^. In our current study, we proved the direct interaction of *P. intermedia* and *T. forsythia* with hDFSCs *in vitro*. Although the adherence of *P. intermedia* to hDFSCs and gingival epithelial cells was indistinguishable, more bacteria entered the dental stem cells, implying a more efficient cell invasion. In fact, little is known about the specific mechanism, but type A fimbriae are suspected to be of general relevance for *P. intermedia* internalization^40^,^41^. A higher number of *T. forsythia* was able to adhere to hDFSCs compared to differentiated Ca9-22 cells, although the internalized bacteria related to the adherent beforehand was significantly higher for Ca9-22 cells. A possible explanation could be the specific expression of surface molecule BspA, which mediates cell adhesion and invasion^40^,^41^,^42^,^43^. Sabet and colleagues described for *T. forsythia* 2% of adherence to and 0.5% of internalization into KB cells^44^, which is comparable to our presented results with regard to a differential experimental set up and MOI. Of note, *P. gingivalis* and *F. nucleatum* show higher affinity towards epithelial cells compared to hDFSCs underlining a possible divergent survival strategy in host tissue compared to *T. forsythia* and *P. intermedia*^18^. The co-infection with a mixture of different oral species may further influence the affinity towards host cells and should be studied in the future.

When challenged with bacterial infection, host cells secrete immunomodulatory cytokines. We have shown here that both, *P. intermedia* and *T. forsythia*, trigger hDFSC IL-6 and IL-8 response, while IL-10 level remain at constant levels throughout the experiment. However, it should be taken into consideration that protease activity can not be excluded and thus influences IL quantification. Guan and colleagues already demonstrated secretion of IL-6 und IL-8 by human periodontal ligament cells after infection with *P. intermedia*, but no stimulation of IL-1ß and TNF-α^19^. *T. forsythia* provoked KB cells to secrete comparable levels of IL-8, but only basal IL-6 levels compared to infected hDFSCs in this study^45^. The IL-8 concentration in the supernatant was lower for *T. forsythia*. Proteolytic enzymes, e.g. putative serine proteases, Karipsin and Karilysinn are identified^46^,^47^ and might influence chemotactic attraction of immune cells. Remarkably, compared to IL-8 secretion levels by hMSCs infected with *Fusobacterium nucleatum*^23^, *P. intermedia* induced a similar phenotype upon hDFSC infection in our study. HDFSCs infected with *P. gingivalis* and *F. nucleatum* are almost unresponsive in terms of IL-8 secretion^18^, a phenotype diverging from hDFSCs infected with *P. intermedia* and *T. forsythia* in this study. This again highlights divergent survival strategies of various oral periodontal pathogens.

Our further results revealed chemotaxis of PMNs towards *T. forsythia* infected hDFSCs is reduced compared to uninfected cells. Of note, the infection did not promote PMN migration. Low IL-8 levels in the supernatant could be a reason for this reduced attraction. In contrast, *P. intermedia* showed increased attraction of PMNs in this experiment. Remarkably, *P. intermedia* infected hDFSCs did not attract immune cells within 2 h post-infection, although IL-8 accumulation was high implying another chemotactic mechanism upon IL-8 response. The constant IL-10 concentration might support this behavior, since it is known to suppress pro-inflammatory cytokine release of PMNs^48^. Another mechanism or so far unidentified secretory factor can be responsible, too.

The immunomodulation of periodontal stem cells was demonstrated by Cianci *et al*.^49^. A specific protective effect of hDFSCs against tissue damage is conceivable and moreover, these effects vary depending on the bacterial species in contact with hDFSCs. Especially remarkable is the reduction of bacterial clearance via PMN killing from the supernatant in the presence of dental stem cells. The bacterial killing by PMNs is more effective in the absence of hDFSCs. Inhibition of direct bacteria-stem cell interaction leads to intermediate clearance. A possible mechanism is the observed bacterial invasion into hDFSCs to evade PMN activity, since the invasion in co-culture without immune cells is negligible compared to the inoculated bacteria. It is known that host defense and PMN activity mediate tissue degradation in periodontitis^50^, thus our results rather suggest a phenotypical environment with strongly reduced tissue and bone destruction.

Main mediators for bacterial recognition are LPS receptor CD14 and toll like receptors (TLRs), e.g. TLR-2 and TLR-4, which play a critical role in periodontal inflammation^51^,^52^,^53^. An induced reduction of host cell CD14 expression by *P. gingivalis* is related to reduced phagocytic activity of macrophages against living bacteria^54^.

The formation of neutrophil extracellular traps was increased upon bacterial challenge compared to infected stem cells, and compared to uninfected hDFSCs. Again, dental stem cells cause a decrease in defense efficiency of PMNs against bacteria.

Generally, PMN activity against oral microorganisms is a complex orchestration of defense strategies, e.g. chemotactic movement to the site of infection, capturing of bacteria in NETs and phagocytic elimination from the environment. HDFSCs apparently protect the oral bacteria against PMN mediated removal, most likely via anti-inflammatory cytokine secretion and internalization of bacteria. Since bacterial survival is increased in the co-culture with local host stem cells, a higher burden of such species is reasonable in periodontal pockets. Otherwise, anti-inflammatory cytokine release prevents against hyperstimulation of PMN and thus aggravated host mediated tissue degradation. This observation with *P. intermedia* and *T. forsythia* is contrary to oral species as *P. gingivalis*, which are known to rapidly increase inflammation^55^. In summary, decreased PMN activity induced by exploitation of hDFSCs by *P. intermedia* and *T. forsythia* could lead to decreases in reactive oxygen species activity and subsequent reduced osteoclast activation, thereby protecting against bone resorption and tooth loss.

Of course, this mono-specific infection is restricted to the *in vitro* model, as complex oral biofilms consists of hundreds of species. On the other hand, mono-causal effects cannot be drawn back in multi-variant experimental setups, and thus undermine better understanding of species-specific interactions.

## Conclusion

Our results pinpoint towards a bacterial species-dependent exploitation of dental stem cells, leading in final consequence to immunomodulatory functions of dental stem cells in bacterially induced periodontal disease. Oral bacteria trigger interleukin response by hDFSCs in *in vitro* co-culture. In the case of *P. intermedia* and *T. forsythia* infected hDFSCs this leads to decreased chemotaxis, phagocytic activity and NET formation of PMNs, implying anti-inflammatory effects of infected dental stem cells. Especially with respect to host tissue damage by activated neutrophils, the role of hDFSCs is a two edged sword, as undermining PMN activity leads to better survival of periodontal pathogenic bacteria, maintaining a constant source of inflammation and immune response. Of note, *P. intermedia* and *T. forsythia* infected hDFSCs maintain their stem cell functionality and archetypical differentiation potential, at the same time signaling towards a reduced PMN mediated innate immune response.

## Methods

### Ethics statements

Ethics approval for all experiments using human materials in this study was granted by the University of Rostock Ethics Committee (https://ethik.med.uni-rostock.de/). All methods using the human stem cells and human blood PMNs were carried out in accordance with all relevant guidelines and regulations and all experimental protocols were approved by the above mentioned Ethics Board.

In detail, human stem cells from the dental follicle (hDFSCs) were isolated from wisdom teeth provided by the Department of Oral and Maxillofacial Plastic Surgery, University of Rostock. Informed consent was obtained from all subjects. Moreover, donors gave their written approval to participate in this study. This procedure was authorized by the ethics committee of the University of Rostock, Germany (Permission No. A 2011 91). Approval for the use and isolation of human polymorphonuclear leukocytes (PMNs) from the blood of healthy volunteers was given by the ethics committee of the University of Rostock, Germany (Permissions No. A 2013 0127 and No. A 2014 0131) and again informed consent was obtained from all subjects.

### Anaerobic cultivation of cells and oral microorganisms

Anaerobic cultivation was realized using the miniMACS anaerobic workstation (Don Whitley Scientific, Shipley, UK) with integrated palladium catalyst, temperature and humidity control. Before entering the workstation two nitrogen flushes were performed to eliminate environmental oxygen. Additionally, absence of oxygen was controlled using anaerobic atmosphere indicator strips (Biomérieux, Marcy-l′Étoile, F).

### Cell isolation and culture

Isolation of human stem cells from the dental follicle (hDFSCs) was performed as described by Haddouti *et al*.^56^ from three young donors with healthy periodontal status. The wisdom teeth were not erupted before extraction to prevent bacterial priming. Cells were cultivated in Dulbecco modified Eagle medium (DMEM F-12, Thermo Fisher Scientific, Waltham, USA) supplemented with 10% fetal calf serum (FCS) and 1% PenStrep (Thermo Fisher Scientific, Waltham, USA) at 37 °C, 5% CO_2_. For experimental setup cells of passages 8 to 12 were used. Living cell count, metabolic activity and expression of stem cell markers were validated before and after co-cultivation of bacteria under aerobic (37 °C, 5% CO_2_) and anaerobic conditions (37 °C, 80% N_2_/10% H_2_/10% CO_2_) at time points 24 h, 48 h and 72 h. The living cell count was determined via trypan blue staining in a Neubauer counting chamber. The metabolic activity was quantified following the MTS based assay protocol (CellTiter96 AQueouse One Solution Reagent, Promega, Madison, USA). Positive expression of CD73, CD29, CD90, CD105 and CD44, and negative expression of CD45 was confirmed. For this, incubated cells were trypsinized, centrifuged (300 g, 10 min, 21 °C), washed with PBS and resuspended in FACS buffer (PBS, pH 7.2, 0.2% BSA). Cells were incubated with antibody and isotype control (Biolegend, San Diego, USA) for 30 min at 4 °C, centrifuged (300 g, 10 min, 4 °C), washed with FACS buffer and analyzed in a flow cytometer (Accuri C6, BD Biosciences, Franklin Lakes, USA).

The human gingival epithelial cell line Ca9-22 was provided by the German Cancer Research Center (DKFZ, Heidelberg, G). Cells were cultivated in Dulbecco modified Eagle medium (DMEM + GlutaMax-I, Thermo Fisher Scientific, Waltham, USA) supplemented with 10% FCS and 1% PenStrep at 37 °C and 5% CO_2_. Cell count and metabolic activity were proved for co-culture conditions as described above.

Human polymorphonuclear leukocytes (PMNs) were isolated from the blood of 8 healthy young volunteers at room temperature. Erythrocytes from heparinized venous blood were lysed by Buffer EL (Qiagen, Venlo, Netherlands), followed by centrifugation (300 g, 10 min, 21 °C) and washing with PBS. Density gradient separation of remaining cells (400 g, 40 min, 21 °C) was performed using Ficoll-Paque PLUS (GE Healthcare Bio-Sciences, Chalfont St Giles, Great Britain). The quality of blood cell separation was assessed via flow cytometry using CD15 antibody (ImmunoTools, Friesoythe, Germany).

### Bacterial species

The strains *Tannerella forsythia* ATCC 43037 and *Prevotella intermedia* ATCC 25611 were acquired from commercial providers (American Type Culture Collection, Manassas, USA), and cultivated on COL agar plates (BD, Franklin Lakes, USA) and in PYG modified with 5 μg/ml hemin and 1% vitamin K_1_ under anaerobic atmosphere (37 °C, 80% N_2_/10% H_2_/10% CO_2_). Growth behavior in cell culture medium DMEM with 10% FCS was validated via optical density at 600 nm in parallel with PYG and BHI and parallel CFU determination of BHI probes on BHI agar plates over 24 h under anaerobic conditions.

### Co-culture

The co-culture of bacteria and human stem cells was established by Kriebel *et al*.^23^. Cells were seeded into well plates without antibiotics and incubated to allow attachment and forming of a 70–80% confluent monolayer at 37 °C, 5% CO_2_. For each co-culture 10^5^ hDFSCs or Ca9-22 cells/ml were used.

Bacterial species *T. forsythia* and *P. intermedia* were grown to stationary phase in PYG modified with 5 μg/ml hemin and 1% vitamin K_1_ at 37 °C, 80% N_2_/10% H_2_/10% CO_2_. Bacterial count was adjusted to 10^7^ microorganisms/ml in DMEM. Adherent human cells were washed with PBS and infected with oral bacteria at a multiplicity of infection (MOI) of 100 in DMEM.

Freshly isolated PMNs were counted using a Neubauer chamber, adjusted to 10^6^ PMNs/ml in DMEM and added to the previous co-cultures. According to the experimental setup, co-culture is realized under different conditions as described below.

### Adherence and internalization

To measure direct interactions of oral bacteria and gingival epithelial cells or hDFSCs, cells were prepared as described in the section co-culture in 24 well plates. Co-culture with the anaerobic bacteria was realized anaerobically (37 °C, 80% N_2_/10% H_2_/10% CO_2_) in DMEM without FCS (MOI = 100). Wells with bacteria mono-culture were used as growth reference.

After 2 h supernatants were removed and cells were washed with PBS. For adherence, human cells were trypsinized, centrifuged (300 g, 10 min, 21 °C), washed with PBS and lysed. Adherent bacteria were counted after plating on COL agar plates. For internalization, fresh medium with 1% PenStrep was added after washing with PBS to kill extracellular adherent bacteria. After a further 2 h incubation period hDFSCs were washed with PBS, trypsinized, centrifuged (300 g, 10 min, 21 °C) and washed with PBS. After cell lysis, internalized bacteria were plated on COL agar.

### Stem cell marker and differentiation

HDFSCs were infected with microorganisms. After 24 h of anaerobic co-culture stem cell surface factors (CD29, CD44, CD73, CD90, CD105 and CD45) and corresponding isotype controls were quantified via flow cytometer as described in section Cell isolation and culture (MOI = 100). Uninfected hDFSCs were used as control under aerobic and anaerobic conditions.

To analyze the influence of infection on differentiation potential of the dental stem cells, hDFSCs were seeded into 24 well plates with cover slips and initially infected with *P. intermedia* or *T. forsythia* for 2 h. Afterwards cells were washed and treated with 1% PenStrep. Uninfected hDFSCs were used as control. After 24 h of aerobic incubation cells were covered with differentiation medium (adipogenic: 1 μM dexamethasone, 0.2 mM indomethacin, 0.5 mM 3-isobutyl-1-methylxanthine, 2 μM Insulin; osteogenic: 1 μM dexamethasone, 10 mM β-glycerophosphate, 50 μM ascorbic acid 2-phosphate; chondogenic: 50 μM ascorbic acid 2-phosphate, 40 μg/ml proline, 100 μg/ml, sodium pyruvate, ITS) with medium exchange after three to four days. Cells with normal cell culture medium DMEM with 10% FCS were used as negative control, uninfected cells in differentiation medium were used as positive control. After 7, 14 and 21 days, cells were stained and analyzed via microscopy. Adipogenic differentiation experimental probes were stained with Oil Red O solution, chondogenic differentiation was visualized with safranin, and osteogenic fate was examined using von Kossa stain.

### Cytokine secretion

HDFSCs and Ca9-22 cells were seeded into 24 well plates and co-cultivated with the anaerobic bacteria in DMEM with 10% FCS (MOI = 100). Uninfected cells served as control. After 2 h, 4 h and 24 h (37 °C, 80% N_2_/10% H_2_/10% CO_2_) supernatants were collected and stored at −20 °C for analysis of IL-6, IL-8 and IL-10 concentrations via OptEIA Human IL ELISA Kits (BD, Franklin Lakes, USA). Performance and calibration were done according to manufacture description. Controls were subtracted from the values determined for infected cells.

### Migration

The ability of hDFSCs to migrate was assessed under aerobic and anaerobic conditions (37 °C). Therefore, a wound closure assay described by Laheij *et al*.^57^ was modified. Cells were seeded into 24 well plates with cover slips. The adherent cell layer was continuously scratched, medium was exchanged and cells were infected with 10^7^ cfu/ml of each bacteria species (MOI = 100). Each scratch diameter was determined in five representative image sections for three technical replicates per experiment each 4 h up to 24 h from the beginning using the Biozero BZ 8000 (Keyence, Osaka, J) and corresponding Biozero software. Uninfected hDFSCs were used as control.

### Chemotaxis

To analyze the chemotactic attraction potential of infected stem cells towards PMNs, the transwell migration assay was modified as described by Nuzzi *et al*.^58^. Transwell permeable support plates (3 μm polyester membrane, Corning, Corning, USA) were coated with 2.5 μg/ml fibrinogen for 1 h and dried overnight. Sterile filtered supernatants of HDFSCs infected with oral bacteria for 24 h in DMEM without FCS (37 °C, 80% N_2_/10% H_2_/10% CO_2_) were used as attractants and added to the lower compartment (MOI = 100). Sterile filtered supernatants of hDFSCs or bacterial mono-cultures were used as controls.

10^6^ freshly isolated PMNs were added into the insert. After 2 h of aerobic incubation the number of PMNs in the lower segments was assessed using trypan blue and a Neubauer counting chamber.

### Phagocytosis

The phagocytosis assay was adapted from Leijh *et al*.^59^. 10^5^ hDFSCs were infected with 10^7^ bacteria in DMEM without FCS (MOI = 100) and 10^6^ freshly isolated PMNs were added. Mono-cultures of bacteria or PMNs were used for control. After 2 h of cultivation (37 °C, 5% CO_2_) PMNs were counted via trypan blue staining and a Neubauer counting chamber, centrifuged (300 g, 10 min, 21 °C) and lysed to determine extracellular and intracellular bacteria and bacterial clearance by PMNs. For control, stem cells were pre-treated for 1 h with Latrunculin B (10 μM) and washed with fresh medium to prevent direct bacteria-stem cell interaction and thus evasion from PMNs.

### NETosis

The formation of NETs was quantified in black 96 well plates after challenging 10^6^ PMNs/ml with 10^7^ microorganisms/ml cultivated with or without 10^5^ hDFSCs/ml (MOI = 100). Control stimulation of PMNs was realized using glucose oxidase (20 mU, from *Aspergillus niger*, Sigma Aldrich). After 165 min of aerobic incubation extracellular DNA was quantified via adding 30 μl Sytox green (50 μM, Life Technologies, Carlsbad, California) to the samples. After 15 min of incubation (37 °C, 5% CO_2_) fluorescence was measured at 485/520 nm.

### Statistics

All experiments were carried out in at least 4 biological replicates. The results are presented as median ± interquartile range. Statistical analysis for significance was realized with Mann-Whitney U test, and significance was defined as *p < 0.05, **p < 0.01 and ***p < 0.001 using the implemented analysis function of GraphPad Prism 6 software (La Jolla, USA).

## Additional Information

**How to cite this article**: Hieke, C. *et al*. Human dental stem cells suppress PMN activity after infection with the periodontopathogens *Prevotella intermedia* and *Tannerella forsythia. Sci. Rep.* **6**, 39096; doi: 10.1038/srep39096 (2016).

**Publisher's note:** Springer Nature remains neutral with regard to jurisdictional claims in published maps and institutional affiliations.

## Supplementary Material

Supplementary Figures and Tables
