# Supplementary material for: Human dental stem cells suppress PMN activity after infection with the periodontopathogens Prevotella intermedia and Tannerella forsythia
Source: Sci Rep. 2016 Dec 15;6:39096. doi: 10.1038/srep39096 (PMC5156907; doi:10.1038/srep39096)
Supplement: Supplementary Figures and Tables [file srep39096-s1.pdf]

Supplementary

Human dental stem cells suppress PMN activity after infection with the periodontopathogens *Prevotella intermedia* and *Tannerella forsythia*

Cathleen Hieke, Katja Kriebel, Robby Engelmann, Brigitte Müller-Hilke, Hermann Lang, Bernd Kreikemeyer

Anaerobic cultivation of hDFSCs

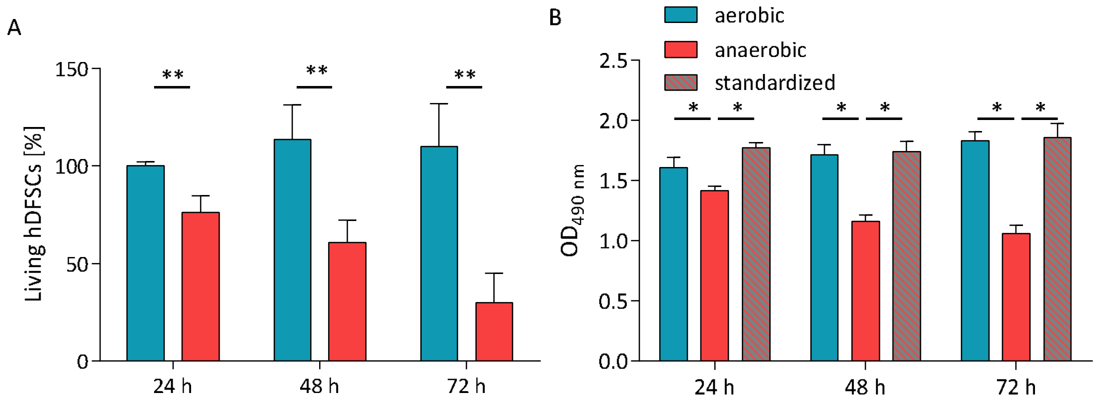

FIGURE S1. Viability of hDFSCs under aerobic and anaerobic conditions. HDFSCs were incubated under aerobic and anaerobic conditions over 72 h. (A) Living cell count was assessed via trypan blue staining, and initial seeded cell count was defined as 100 %. (B) Metabolic activity of hDFSCs was quantified via MTS assay. Aerobically incubated cells are presented in blue, anaerobically incubated in red. Standardization of anaerobic metabolic activity was adapted to cell count. Results are displayed as median ± interquartile range, **p < 0.01 (Mann-Whitney U test), n = 4.

Table S1. Median fluorescence intensity (MFI) of stem cell markers of hDFSCs under aerobic and anaerobic conditions over 72 h. Isotype antibodies were used as negative control. Delta MFI was calculated to quantify change in fluorescence, thus confirmation of specific stem cell surface markers. Means are represented in bold digits with standard deviation. Low or negative values refer to unspecific binding only.

ΔMFI	24 h, aerobic	24 h, anaerobic	48 h, anaerobic	72 h, anaerobic
CD45	-4.953	-1.081	-3.151	787
	-2.686	-2.619	79	-2.308
	-1.230	-1.300	-193	-1.781
	**-2.956 ± 1.532**	**-1.667 ± 679**	**-1.088 ± 1.463**	**-1.101 ± 1.352**
CD90	1.041.705	1.095.231	981.508	886.359
	988.051	1.062.889	815.566	705.104
	774.663	997.483	781.962	675.956
	**934.806 ± 115.337**	**1.051.868 ± 40.659**	**859.679 ± 87.232**	**755.806 ± 93.078**
CD73	885.963	845.160	880.360	797.578
	756.056	711.949	880.041	791.543
	695.692	592.273	878.576	775.213
	**779.237 ± 79.389**	**716.461 ± 103.290**	**879.659 ± 777**	**788.111 ± 9.447**
CD105	68.481	76.646	66.429	60.694
	64.193	73.050	54.410	45.647
	48.661	68.771	53.223	42.217
	**60.445 ± 8.514**	**72.822 ± 3.219**	**58.021 ± 5.965**	**49.519 ± 8.025**
CD44	2.994.854	2.769.185	1.788.062	1.332.839
	2.088.482	2.756.392	1.116.347	883.018
	1.678.236	1.027.216	1.082.476	861.575
	**2.253.857 ± 550.080**	**2.184.264 ± 818.173**	**1.328.962 ± 324.927**	**1.025.811 ± 217.278**
CD29	639.369	496.870	406.992	391.755
	404.232	449.009	322.007	287.410
	293.875	302.561	317.601	274.942
	**445.825 ± 144.081**	**416.147 ± 82.660**	**348.867 ± 41.140**	**318.036 ± 52.375**

Table S2. Median fluorescence intensity of stem cell markers of hDFSCs infected with *P. intermedia* and *T. forsythia* under anaerobic conditions after 24 h. Isotype antibodies were used as negative control. Delta MFI was calculated to quantify change in fluorescence, thus confirmation of specific stem cell surface markers. Means are represented in bold digits with standard deviation. Low or negative values refer to unspecific binding only.

ΔMFI	hDFSC	hDFSC + *P. intermedia*	hDFSC + *T. forsythia*
CD45	-1.081	2.410	-1.636
	-2.619	3.554	1.083
	-1.300	-832	-899
	**-1.667 ± 679**	**1.711 ± 1.858**	**-484 ± 1.148**
CD90	1.095.231	893.521	969.025
	1.062.889	775.896	826.676
	997.483	400.189	800.328
	**1.051.868 ± 40.659**	**689.869 ± 210.388**	**865.343 ± 74.099**
CD73	845.160	643.863	792.796
	711.949	592.483	716.421
	592.273	557.687	666.360
	**716.461 ± 103.290**	**598.011 ± 35.398**	**725.192 ± 51.989**
CD105	76.646	49.712	53.529
	73.050	49.869	54.021
	68.771	29.269	53.149
	**72.822 ± 3.219**	**42.950 ± 9.674**	**53.566 ± 357**
CD44	2.769.185	984.738	1.198.686
	2.756.392	743.488	1.137.995
	1.027.216	466.639	872.046
	**2.184.264 ± 818.173**	**731.622 ± 211.679**	**1.069.576 ± 141.855**
CD29	496.870	355.390	370.932
	449.393	323.701	337.347
	302.177	161.813	283.353
	**416.147 ± 82.887**	**280.301 ± 84.777**	**330.544 ± 36.076**
